# The formation of araneiforms by carbon dioxide venting and vigorous sublimation dynamics under martian atmospheric pressure

**DOI:** 10.1038/s41598-021-82763-7

**Published:** 2021-03-19

**Authors:** Lauren Mc Keown, J. N. McElwaine, M. C. Bourke, M. E. Sylvest, M. R. Patel

**Affiliations:** 1 Trinity College Dublin, College Green, Dublin 2, Ireland; 2 Durham University, Durham, DH1 UK; 3 Planetary Science Institute, 1700 E Fort Lowell Rd, Tucson, AZ 85719 USA; 4 The Open University, Walton Hall, Milton Keynes, MK7 6AA United Kingdom; 5 Present Address: The Open University, Walton Hall, Milton Keynes, MK7 6AA United Kingdom

**Keywords:** Cryospheric science, Inner planets

## Abstract

The local redistribution of granular material by sublimation of the southern seasonal ${\hbox {CO}}_2$ ice deposit is one of the most active surface shaping processes on Mars today. This unique geomorphic mechanism is hypothesised to be the cause of the dendritic, branching, spider-like araneiform terrain and associated fans and spots—features which are native to Mars and have no Earth analogues. However, there is a paucity of empirical data to test the validity of this hypothesis. Additionally, it is unclear whether some araneiform patterns began as radial and then grew outward, or whether troughs connected at mutual centres over time. Here we present the results of a suite of laboratory experiments undertaken to investigate if the interaction between a sublimating ${\hbox {CO}}_2$ ice overburden containing central vents and a porous, mobile regolith will mobilise grains from beneath the ice in the form of a plume to generate araneiform patterns. We quantify the branching and area of the dendritic features that form. We provide the first observations of plume activity via ${\hbox {CO}}_2$ sublimation and consequent erosion to form araneiform features. We show that ${\hbox {CO}}_2$ sublimation can be a highly efficient agent of sediment transport under present day Martian atmospheric pressure and that morphometry is governed by the Shields parameter.

## Introduction

The Martian surface is host to a variety of features which have no Earth analogs and are all active in some capacity today. These include the ephemeral sand furrows^1^ which seasonally scour the surface of Martian dunes, only to be later erased by aeolian activity, the enduring and growing dendritic troughs of the southern mid-to-high latitudes^2^ and the ‘spider-like’ araneiform terrain of the south polar cryptic region^3^ from which seasonal fans and spots frequently emanate^4^. All of these active geomorphological features exhibit dendritic patterns. These patterns are fractal, resemble tree branches and are found on Earth in systems that are engendered by physical gradients, such as river networks, fork lightning strikes and even nerve endings in the human brain.

On Mars, dendritic patterns are emblematic of exotic geomorphological processes. These processes are driven by a cycle which does not occur on Earth; namely the seasonal phase change of carbon dioxide between solid and gas. The Martian atmosphere comprises over 95% ${\hbox {CO}}_2$^5^, at least a quarter of which freezes each year^6^. As winter comes and temperatures decrease, a dry ice layer is deposited on the Martian surface^6^ ranging in thicknesses of nearly a metre at the poles, to a few millimetres further towards the equator^7,8^. The average atmospheric pressure on Mars is very low at 6 mbar, below the triple point and so as this ice heats in spring, it sublimates; that is, it directly converts from solid to gas. The basal sublimation of the seasonal cap is now recognised as a cardinal geomorphological agent responsible for reworking the surface of Mars today^9^, in particular forming araneiforms, which are unlike any surface feature seen on Earth.

**Figure 1:**
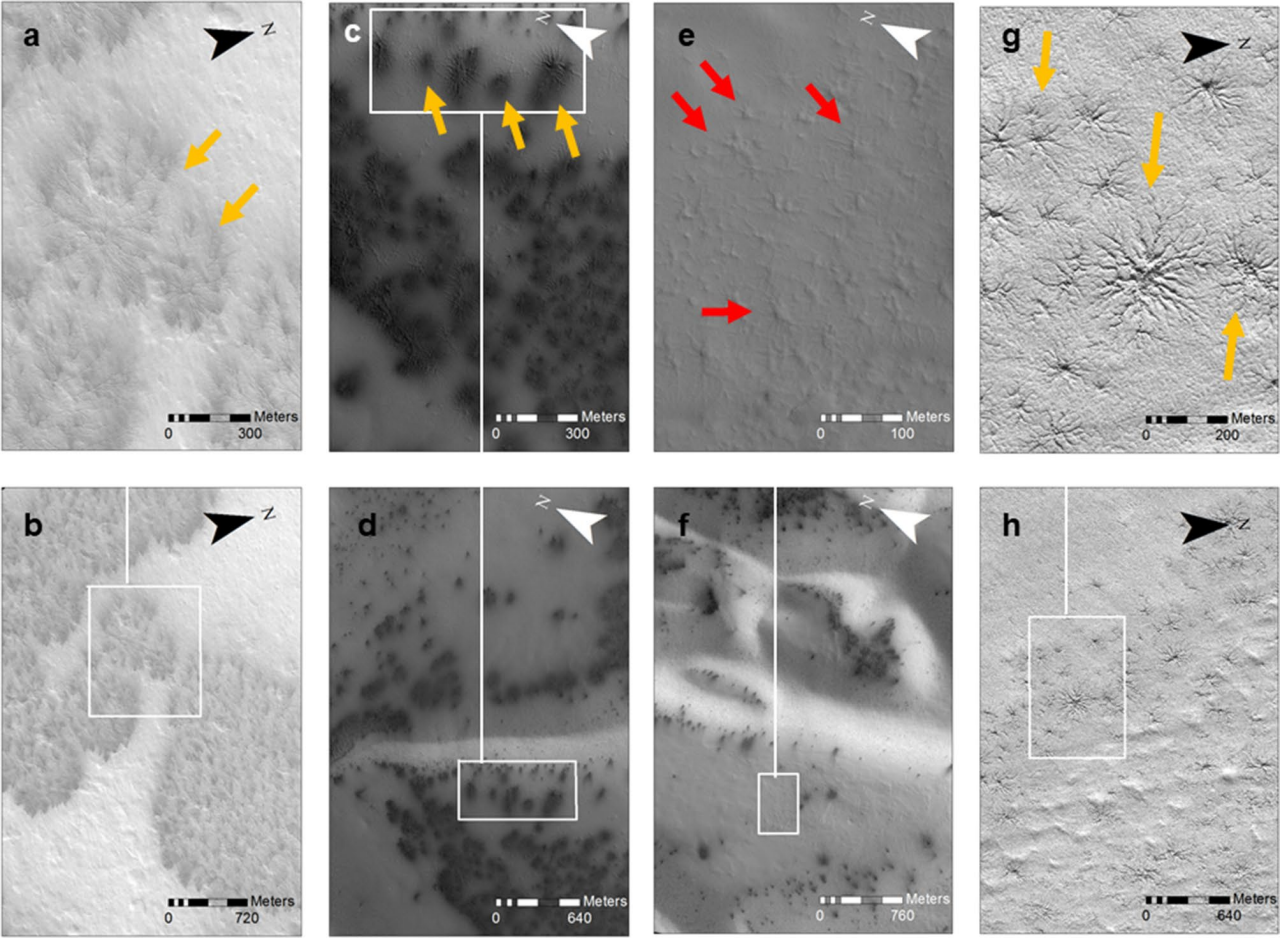
Zoomed and corresponding context images of a variety of araneiform morphologies (**a**, **b**) HiRISE image $ESP\_011486\_0980$ shows ‘Starburst araneiforms’ which are huge features. Central lat $=-81.8^{\circ }$, Central lon $=76.17^{\circ }$, $L_s=187.3^{\circ }$. (**c**, **d**) HiRISE image $ESP\_011491\_0985$ shows seasonal dark albedo fans and spots. Central lat $=-81.2^{\circ }$, Central lon $=296.04^{\circ }$, $L_s=187.5^{\circ }$. (**e**, **f**) shows ‘fat spiders’ in HiRISE image $ESP\_014282\_0930$. (**g**, **h**) shows close ups of ‘classic’ spiders in the same image as (**e**, **f**) only a different location within the site. Central lat=$-87.02^{\circ }$, Central lon $=86.57^{\circ }$, $L_s=320.5^{\circ }$. HiRISE image credit: NASA/JPL/University of Arizona.

Araneiforms (Fig. 1), frequently dubbed “spiders”, are characterised by a central depression from which radial tortuous troughs appear to emanate^3^. These negative topography radial troughs become shallower further away from the central depression and have dendritic and tortuous network patterns. In spring, araneiforms are frequently accompanied by relatively low albedo fans and spots (Fig.1c,d)^3,9,10^ which appear on top of the seasonal ${\hbox {CO}}_2$ ice layer that covers them. Araneiforms are observed mainly on the Cryptic Terrain of the South Polar Layered Deposits (SPLD)^3^ and upon neighbouring crater blankets^11,12^. They usually occur in groups, sometimes packed densely enough to overlap^3^ and are generally found on low surface slopes^13^. While the central depression of a single araneiform is typically $\sim$ 50 m across, the entire feature including its troughs spans from tens of metres to up to 1 km across^3,13^. A survey of araneiforms at the Inca City and Manhattan regions of the SPLD allowed a sub-classification of these features according to morphometry^13^. This classification identified araneiforms based on whether their patterns were radial or not, on the scale of channels relative to the central depression and on the tortuosity of the channels. “Fat spiders” (Fig. 1e,f) consist of wide central depressions with respect to radial troughs^13^ and are generally smaller. These spiders measure $\sim \,50\,$m across with 30–40 m of that spanning their centres. “Thin spiders” (Fig. 1g,h) have large, rough centres with long, thin troughs radiating outwards and generally cover more area ($\sim \,100\,$m across). “Lace terrain” describes dense networks of channels similar in size where a singular radial spider morphology cannot be distinguished^13^ and “starburst” araneiforms (Fig. 1a,b) are highly dendritic with thousands of branches, and can extend up to $1\,$km in diameter. These are all distinct species which are each located in different regions^14^.

Araneiforms have been attributed to the erodability of the surface material of the SPLD which is estimated to contain silt-sized particles 50–200 $\upmu$m in diameter^15,16^. The discovery that the fans and spots were appearing in a ‘Cryptic Terrain’, or region which remains at the same brightness temperature as ${\hbox {CO}}_2$ ice^9^ in spring despite a decreasing albedo, led Kieffer to propose that the seasonal fans and spots were not exposed terrain as previously thought^17^, but in fact excavated sub-ice material transported on top of the ice by confined gas beneath the seasonal ${\hbox {CO}}_2$ layer. Invoking the Solid State Greenhouse Effect^18^, whereby broad spectrum radiation infiltrates translucent ice to reach the subsurface, the model asserts that in spring, solar radiation penetrates the seasonal translucent slab ice and heats the regolith beneath it. Thermal radiation cannot escape through the ice overburden because it is opaque at thermal infrared wavelengths and so the ice will sublimate from the base. As pressure increases, at some point the ice will rupture and form a vent. Gas will escape through this vent, drawing with it unconsolidated fines from beneath the ice in the form of a plume, and depositing the material as relatively low albedo fans and spots. A combined effort by Piqueux^3^ who identified and described spiders, and Kieffer, who developed a formation hypothesis, linked araneiform formation with the seasonal appearance of fans and spots. Their dendritic negative topography patterns are thought to be eroded by pressurised escaping ${\hbox {CO}}_2$ gas^3,9^. That the seasonal ${\hbox {CO}}_2$ deposit anneals annually into translucent slab ice has been confirmed by Mars Express Omega observations^19^ and it has been calculated that 75% of incident solar radiation will reach the regolith beneath the ice overburden^20^. However, although laboratory experiments have generated araneiform patterns on thin films of sand using a Hele-Shaw cell^21^, particles entrained in jets under Martian conditions have been successfully modelled^22^ and dust ejecta have been observed from within irradiated ${\hbox {CO}}_2$ ice^23^, a ${\hbox {CO}}_2$ venting process has never been observed to form araneiforms.

Many other open questions remain surrounding araneiforms and their proposed formation mechanism. Firstly, the seasonal appearance of fans and spots is to date the only notable activity associated with araneiforms. Additionally, despite observational campaigns to capture stereographic images of plumes^13,22^, a plume in action has not yet been observed. This is either because the plumes are shallow, optically thin, or their activity has not coincided with HiRISE flybys. While the troughs are thought to marginally widen and deepen annually^13^, the Mars Reconnaissance Orbiter High Resolution Imaging Experiment (HiRISE) which has a resolution of 50 cm^24^, has not been able to detect changes in the araneiforms^2^ over the last 7 years of monitoring. Moreover, the large developed araneiforms of the SPLD have not yet been observed to *form* in the present day. This either means that these araneiforms are growing at a very slow rate and this change has simply not been observed yet, or present day atmospheric conditions and/or sediment consolidation are not conducive to erosion of the substrate.

However, due to the vast volume of material (on the order of $10^3\,{\text {m}}^3$)^13^ that would need to be eroded in one process and hence the requirement of an extremely high energy budget, it is assumed that these patterns required multiple erosional events to form. On this premise, estimates have been made to date the age of araneiforms to $1\times 10^3$–$1\times 10^4$ Mars years (MY)^2,25^. The most recent effort at dating araneiforms was based on the idea that dendritic trough growth might showcase the initial stages of araneiform development. Dendritic troughs are a recent class of negative topography, dendritic feature exclusive to the Martian southern hemisphere and are generally found on dune boundaries^2^. They endure seasonally and have been observed to extend and merge over the last 5 MY^2^. However, how gradually growing troughs would eventually develop into the large, currently apparently inactive araneiforms has not been fully delineated. It is likely that the substrate at dune boundaries is more erosive in the present day, as suggested by Portyankina^2^. However, there are many other variables that may influence araneiform development; for example grain size, sediment consolidation, ice thickness, vent spacing and size and how current atmospheric conditions balance with these factors to result in sufficient erosion of the substrate. It is possible that araneiforms did develop over time via a connection of tributaries as a central depression may continually provide a point of weakness for the ice to rupture^14^. However, this does not explain why new troughs have not yet been observed to form within an existing radial araneiform in the present day. Whether the araneiforms of the SPLD were formed through connecting tributary systems over many years, or whether they are relic features formed in different climatic period(s), remains uncertain. Insights have been given through our prior experiments indicating that grain size and vent separation can influence cryoventing and subsequent patterns^26^, as well as through observations of clustering araneiform sub-types on Mars suggesting grain size and sediment consolidation may play a role in morphology type^14^. However what factors govern the differing araneiform morphologies, their varying levels of branching or the extent of the areas they cover have not been fully elucidated.

Empirical observations are imperative to answering these open questions surrounding araneiforms, though there have been few experiments undertaken to explore ${\hbox {CO}}_2$ sublimation under Martian atmospheric conditions^23,26–28^. Our previous experiments^26^ indicated that ${\hbox {CO}}_2$ gas velocity controlled furrow network pattern and that dendritic morphologies scaled positively with greater vent spacing^26^. We hypothesise that just as furrow formation is governed by high gas velocities, araneiform formation will be too. Suppose a series of vents forms and for a particular vent all the gas from within an area *A* escapes through that vent. If the sublimation rate under the ice is roughly constant and equal to *q* then the total volume flux though a vent will be *Aq* giving rise to gas velocities into the vent of $U=Aq/a$ where *a* is the cross-sectional area of the vent. Since *A* will increase as the separation between the vents increases the gas velocities will also increase. The ability of a gas flow to move a grain is given by the Shields parameter

1 $$\begin{aligned} S=\frac{\rho _g U^2}{\left( \rho _s-\rho _g\right) D g}, \end{aligned}$$

where *g* is gravity, *D* the grain diameter, $\rho _s$ the grain density, $\rho _g$ the gas density. (This is in the case of high particle Reynolds number though the conclusions are the same for low particle Reynolds number). When *S* is above a critical value grains can be mobilised, thus as *A* increases, hence *U*, more grains will be moved but as the particle diameter *D* increases fewer grains will move. We hypothesise based on our previous experiments that the more mobile grains are the more branched the pattern is likely to be.

## Methods

In order to test the hypothesis that radial dendritic araneiform morphologies can form via basal sublimation of ${\hbox {CO}}_2$ and consequent plumes, and to answer some of the open ended questions surrounding the physical constraints on araneiform formation, experiments involving the placement of ${\hbox {CO}}_2$ blocks containing central vents on granular surfaces were performed under Martian pressure. We herein refer to this type of experiment as phase 1. As in our previous Earth-based experiments which explored furrow formation^26^, the basal heating of ${\hbox {CO}}_2$ ice was modelled by exploiting an effect similar to the *Leidenfrost Effect*^29^, as in the work of Diniega et al.^30^. This is an effect that occurs when a substance (in our case solid ${\hbox {CO}}_2$), in contact with a surface far hotter than its boiling or sublimation point, will generate a cushion of high pressure vapour. The gas pressure of this vapour layer exceeds the pressure exerted by the weight of the ${\hbox {CO}}_2$ ice, allowing it to levitate. The point at which solar radiation heats the substrate beneath seasonal ice on Mars enough that it will cause the ice to sublimate from its base can hence be approximated, albeit on a much smaller scale and with different levels of energy.

In order to study the influence of grain size and vent diameter as well as vent separation on feature morphometry, the area araneiforms cover and the level of branching, Digital Elevation Models (DEMs) of the surface features were generated by Structure from Motion (SfM)^31^. Feature dimensions were measured in ArcMap 10.4 and these data were analysed using Matlab R2018a.

The efficiency of ${\hbox {CO}}_2$ sublimation as an agent of sediment transport under Martian pressure was investigated by calculating sublimation mass flux of ${\hbox {CO}}_2$ and estimating sediment displacement per volume of subsurface ${\hbox {CO}}_2$. We herein refer to experiments of this nature as phase 2.

### Experimental setup

**Figure 2:**
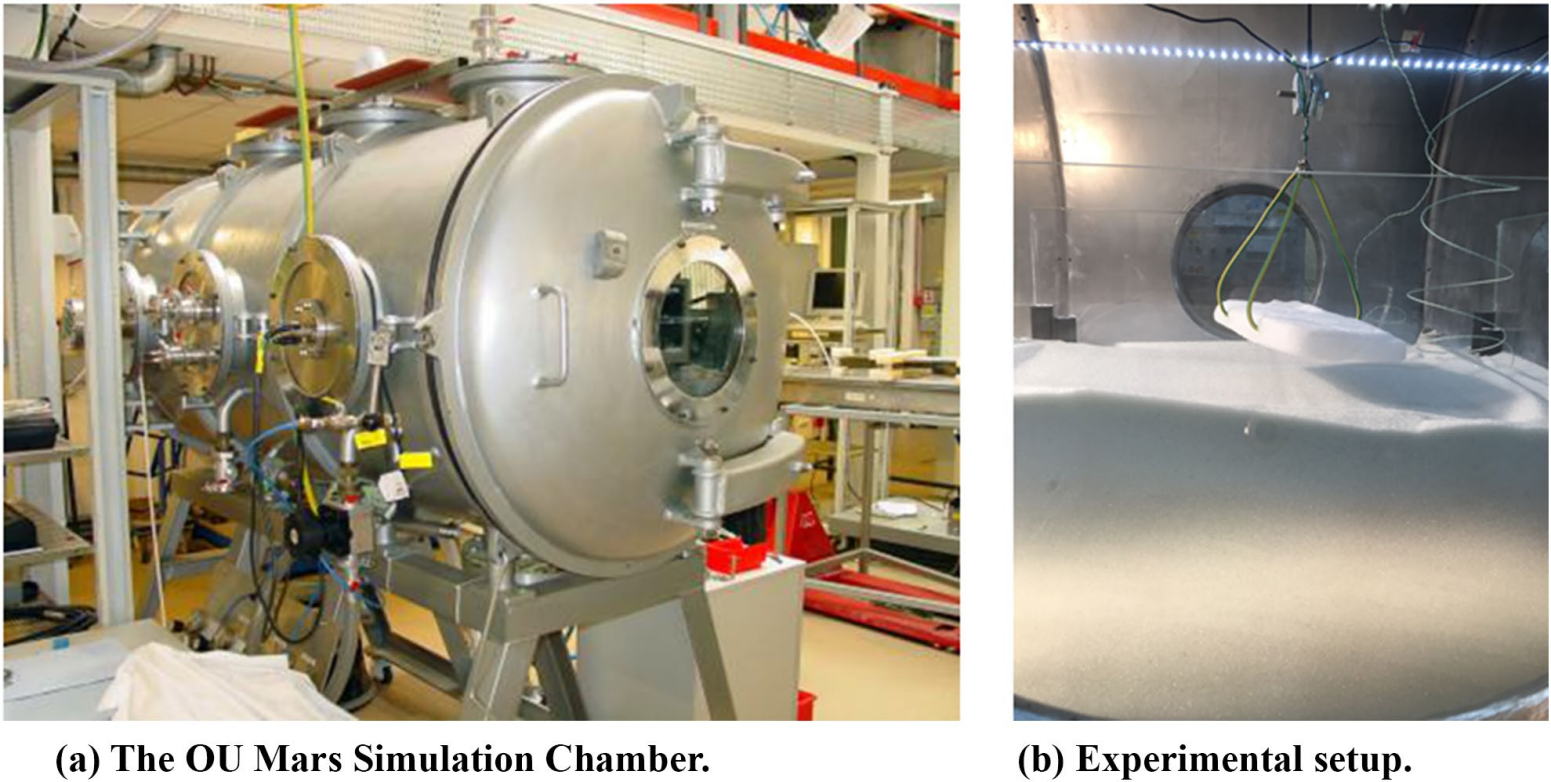
Experimental setup. (**a**) shows the Mars Simulation Chamber which is capable of simulating Martian atmospheric parameters. Image credit: The Open University. (**b**) shows a ${\hbox {CO}}_2$ block suspended above a granular bed with a claw which is slotted into its sides.

The Mars Simulation Chamber (Fig. 2a) at the Open University is capable of recreating atmospheric parameters characteristic of the Martian environment. The chamber measures 0.9 m in diameter and 1.8 m in length and can depress internal pressure to < 6 mbar in less than 5 minutes. A glass aquarium was deployed on a flat track within the chamber and two Logitech webcams were positioned at both a nadir and side view of the tank. In each experimental trial, the tank was filled up to a height of $\sim \,25\,$cm with Guyson Honite glass spheres of distinct grain size ranges. These were chosen due to their unimodal nature which is helpful when developing numerical models. For phase 1 experiments, manufacturing dispersion grain size ranges chosen were 160–212 $\upmu$m, 150–250 $\upmu$m, 250–425 $\upmu$m and 425–600 $\upmu$m. For phase 2 experiments, the smaller two grain size ranges were used. The smaller grain sizes were used to explore the maximum efficiency of ${\hbox {CO}}_2$ sublimation for dust sized particulate matter. A thermocouple was placed within the bed while one was placed outside of it in each case in order to monitor temperature. However this was only indicative of temperature away from the block in real time. Utilising data from extra pilot experiments we performed of thermal gradient measurements beneath the block, we report that initial bed temperature beneath the block was between 16 and 18 $^{\circ }$C and temperature decreased exponentially over time to 0 $^{\circ }$C in just under an hour. Pressure within the chamber was monitored carefully in real time with a pressure gauge.

In order to establish scale in the DEMs, coded targets were printed and attached to plastic poles so that they could be seen at different heights above the sand bed. This was to avoid wide uncertainties in calculation of the z-plane in the 3D model. Their positions in x, y and z were measured with a metal ruler and to improve accuracy, photographs were taken of their positions relative to the mm markings on the ruler. The photos were zoomed so that sub-mm position accuracy could be achieved. A pyramid-shaped claw was developed and attached to an external pulley (Fig. 2b). The pulley was given enough slack so that the claw would just touch the bed surface upon lowering and would be well above the bed when retracted. This was to allow control over timing of block contact with the bed surface and also to ensure the block could be held in place so it would not burrow and erase surface microtopography.

### Experimental protocol

For each experiment, the glass spheres were levelled using a spirit level and smoothed with a metal ruler. Two holes were drilled on one of the longest sides of a ${\hbox {CO}}_2$ ice block and one was drilled on the opposite side so that the claw could be slotted into the block. The holes were drilled close to the bottom of the block so that the claw would not sear through the block fully and hence detach the block prematurely while the experiment was taking place. For phase 1 experiments, holes were created at the centres of the blocks by drilling through fully with different sized drillbits. To investigate the effect of vent dimension and spacing on araneiform morphometry, three scenarios were explored: a central hole measuring 5 mm in diameter, a central hole measuring 3 mm in diameter and two holes along the long axis of the block and at the centre of the short axis, measuring 3 mm in diameter and 60 mm apart. In phase 2 experiments, only holes at the sides of the block were generated in order to insert the claw. All experiments were performed in duplicate.

In each case, the block was weighed using a digital scales and its dimensions were measured with a metal ruler. The time of measurement was noted. The claw was slotted into the block and the block was suspended from the pulley above the bed surface (Fig. 2b). The chamber was sealed, depressurised and when ambient pressure reached 6 mbar, the block was gently lowered onto the surface using the pulley. The time between block measurement and contact with the granular surface exceeded no more than $\sim$ 5 min for each experiment. Our measured rates of ${\hbox {CO}}_2$ sublimation under Mars and Earth pressures were averaged to account for the amount of ${\hbox {CO}}_2$ that was likely to have sublimated during chamber depressurisation. This was subtracted from the measured mass in each case to estimate block mass upon surface contact. The chamber pressure was monitored so that it did not drastically deviate from average Martian atmospheric surface pressure. The chamber is difficult to maintain at an exact low pressure for these types of experiments however because the pumping rate needs to match volatile evolution in order to remain constant and so this fluctuated, but did not deviate by more than 3 mbar in any case. In phase 1 experiments, the block was lowered so that it was sitting on the bed surface, but also held in place by tension in the pulley. The block was allowed to sublimate for $\sim$ 5 min and was then raised very carefully above the bed surface. This was to avoid the block detaching and burrowing, hence erasing surface topography generated by the venting process, and also to avoid spilling granular material from the top of the block on to the surface feature generated. This proved particularly precarious for the finest grain sizes of 160–212 $\upmu$m as the block transported material most rapidly for finer grain sizes.

For phase 2 experiments, the block was lowered and the pulley was left slack to allow the block to detach from the claw as it sublimated and burrowed within the granular bed. Once detached, the claw was raised while burrowing continued. Once sublimation had ceased (i.e. the bed was observed to be still and no granular ejecta were observed), the chamber was repressurised and the bed surface was imaged at many overlapping positions to collect data for DEM production.

### Digital elevation model development

Both the araneiform features and volume change resulting from burrowing were modelled in three dimensions by SfM using Agisoft Photoscan. SfM is a technique for reconstructing three dimensional structures from a series of two dimensional images. Agisoft Photoscan is commercially available software which can photogrammetrically process digital images to create a 3D reference frame. Each feature produced was imaged at many overlapping positions. For ground control, coded markers were placed within the scene. Agisoft Photoscan finds the exact centre of coded markers enabling the production of highly accurate DEMs and the accurate measurement of features in the scene. Agisoft recommend that three or more scale bars are optimal. Therefore, a local coordinate system composed of seven coded markers at known distances and heights apart from one another, was used to establish scale. Black and white circular 12-bit coded markers 30 mm in diameter were printed on paper and fixed upon plastic poles which were stuck securely to the container floor with silicone. The (x, y, z) coordinates of the marker centres were carefully measured with a metal ruler and photographs of their positions were taken. The photographs were zoomed and accurate positions were noted. These were later entered in Agisoft Photoscan to develop scale bars for reference within the models. Their coordinates (in metres) were: (0.016, 0.014, 0.2745), (0.0455, 0.013, 0.2595), (0.343, 0.013, 0.2585). (0.3685, 0.0145, 0.2775), (0.3695, 0.5465, 0.258), (0.3685, 0.5710, 0.2725), (0.0135, 0.5425, 0.26), (0.013, 0.5705, 0.298) and these have accuracy $< 1$ mm. The targets were close enough to the bed centre so that they could be seen in multiple overlapping images. Constancy of target position was assured by the remote nature of the laboratory—external vibrations were minimised and the aquarium containing sediment was heavy enough that it was extremely difficult to move within the chamber.

The images were captured with an iPhone 6s 12 megapixel camera. Images were taken at a maximum distance of $< 1$ m from the bed surface and minimum distance of $\sim 0.1$ m at a variety of angles with respect to the image subject in each case. Camera positions were not recorded, as Agisoft Photoscan can compute accurate estimates and ‘Location Services’ were disabled in order not to interfere with position interpolation in the model. The focal length on the camera and aperture were fixed at 4.15 mm and f2.2 respectively and otherwise, the camera was not calibrated. Between 30 and 70 images were captured for each experiment depending on the level of detail to be recorded. The images were then aligned and referenced in Agisoft Photoscan, to build a point cloud, mesh and (after extraneous dense cloud data were removed), generate a DEM and corresponding orthophoto of each feature. The dimensions of each feature were then measured in ArcMap 10.4, using the DEM and orthophoto.

The depth, width and area of araneiform features and their morphological characteristics produced on each bed surface were recorded using polygonal mapping in ArcMap 10.4. Width was calculated using the ‘Measure’ tool and taking the average of five cross-sections across a similar region. Depth was calculated using the ‘Interpolate Line’ tool, identifying the difference between the highest and lowest point on each side of the resultant plot and averaging these, then taking an average of five of these cross-sections across a similar region. Area rather than volume was measured as most of the araneiforms were sub-DEM resolution in depth and approximating would introduce considerable error. The area of the flat pit floor in each case was determined by zooming in to optimal pixel resolution on the orthophoto overlain above the DEM and using the free-hand polygon tool to mark the line where the inner slope of levées ended and the flat pit floor began. The pit floor is defined as the reasonably flat area directly below where the incident block was for which the perimeter is identified as the line between where the inner levée slope ended at 1 pixel resolution. Levées are defined as the positive relief material surrounding the pit which was excavated and pushed to the sides of the block during sublimation. The area of the space between araneiform networks was determined by zooming in to optimal pixel resolution and mapping the outer edge of each araneiform leg which was identified as where a region of low albedo (negative topography) ended and high albedo (positive relief) began. This total area was differenced from the total pit floor area to get the area covered by araneiform patterns. This was then expressed as a percentage of pit floor area.

**Figure 3:**
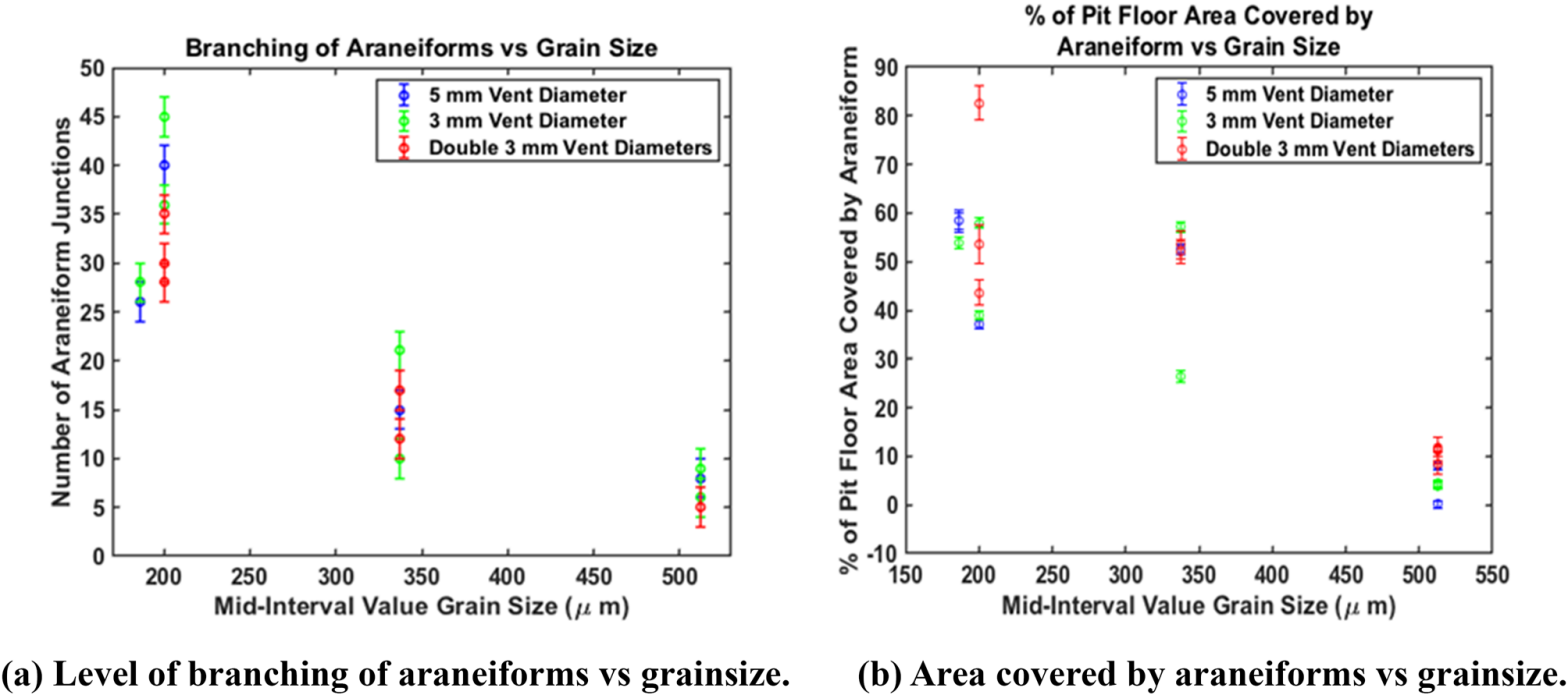
Araneiform morphometry. (**a**) Shows a decrease in the level of branching with grain size. Level of branching varies for finer grain sizes while for coarser grains of 450–600 $\upmu$m, is consistent. (**b**) Shows a decrease in area covered by araneiforms with grain size.

The error bars seen in Fig. 3 were calculated considering the factors that contributed to measurement uncertainty. Firstly there are errors due to imprecision in construction and measurement of our calibration targets. The construction of the objects was much more accurate than the measurement of their locations which we estimate as better than $1\,$mm. Additionally, some of the targets were covered with sediment during the sublimation process making it difficult to pinpoint their exact centres. Though estimated to be minimal, external vibrational disturbances cannot be entirely accounted for. Additionally, despite zooming to one pixel to take our vertical and horizontal measurements, operator error may have occurred. Having tested our approach by making repeat measurements, we estimate the contribution of this error to be $\pm \,3$ pixels in each case, or $\pm \,9$ pixels for each dimension squared. In considering all of these errors, an estimate of 10 pixels was used to cover uncertainty when calculating the area covered by araneiforms. This was propagated in the formula for percentage area using standard error propagation.

**Figure 4:**
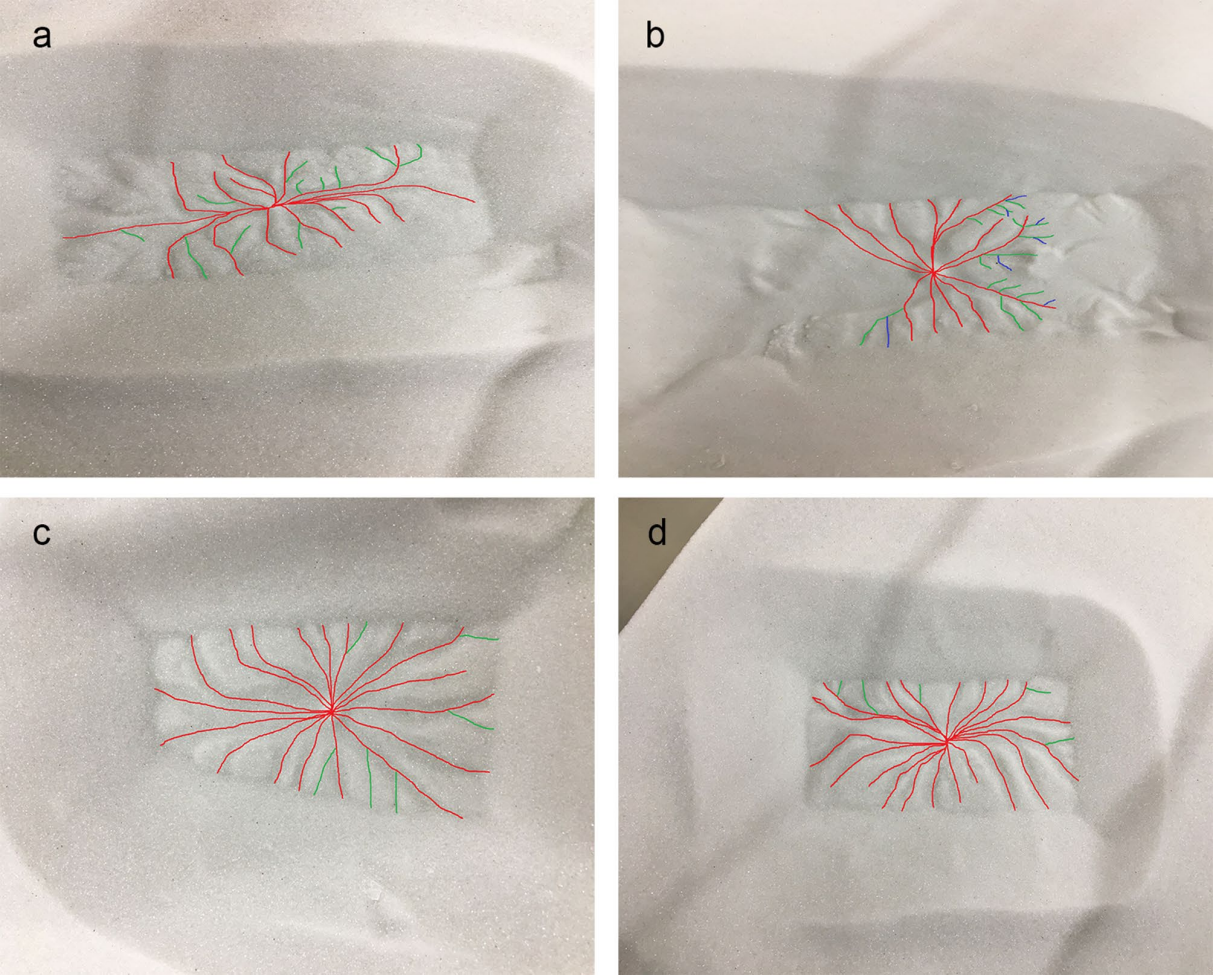
Level of branching and vent diameter. Red shows first order branches, green shows second order and blue shows third order. Each joining of a colour represents a ‘junction’. (**a**) Shows an araneiform formed with a 3 mm vent on 250–425 $\upmu$m grains. 12 junctions are counted. (**b**) Shows an araneiform formed with a 3 mm vent on 160–212 $\upmu$m grains. 22 junctions are counted. (**c**) Shows an araneiform formed with a 5 mm vent on 160–212 $\upmu$m grains. Six junctions are counted. (**d**) Shows an araneiform formed with a 5 mm vent on 250–425 $\upmu$m grains. Five junctions are counted. Level of branching increases (1) when smaller central vents were used and (2) with smaller grain sizes. A more diffuse, steady plume was observed for 5 mm vents while a high velocity, higher plume was observed for 3 mm vents.

The level of branching of araneiforms (Fig. 4) was determined by counting the amount of ‘junctions’ on each araneiform. A junction is defined using the Strahler Stream Order method^32^, (where all streams begin as order 1 (red)) as either an order 1 stream meeting an order 2 stream (green), or an order 2 stream meeting an order 3 stream (blue). Any araneiforms which were partially covered by sediment which fell from the suspended block after sublimation were not analysed in this way and were not included on the plots in Fig. 3 for consistency. An uncertainty of two branches was used in each case as though care was taken, operator error may have mistaken image artefacts for very small branches.

For phase 2 experiments, sediment volume displacement was estimated. Differencing before and after DEMs was not possible due to the high albedo of the granular surface which is difficult to capture as a 3D model when flat. Additionally, due to bright regions on the relatively gentle slopes left after the block had burrowed, DEMs contained holes in some places. Thus, generating a planar surface in ArcMap 10.4 under which to interpolate volume would reduce the accuracy of our volume calculations.

Instead, because the surface was smoothed and levelled prior to each experiment, the initial height of the levelled glass spheres in the container was noted. The initial volume was calculated as width $\times$ length $\times$ height of the bed in each case. In all four cases, dips in the bed were visible. Regions of similar depth were identified and the average depth for each region was noted by interpolating a line across the dip and subtracting the average maximum depth from the original height of the unperturbed bed. A polygon tool was then used to mark the outer edges of these individual regions and the area they covered was calculated. These areas were then multiplied by average depth and summed together in order to get an estimate of the volume displaced during the burrowing process. In cases where the moving block had increased the sediment height in locations, the difference between the original bed height and new height were multiplied by area. This method, while approximate, avoided incorporating bright regions with holes into volume calculations which could introduce order of magnitude uncertainties into our volume estimates.

Sublimation mass flux was calculated by measuring the mass of the ${\hbox {CO}}_2$ block before and after sublimation and burrowing. Activity due to burrowing was taken as complete once no movement was observed within the bed. If any ${\hbox {CO}}_2$ ice remained within the bed post-burrowing, this ice was removed and weighed. Sublimation time was taken as the time between block contact with the surface and the time activity was observed to cease. This time was recorded via webcam. Sublimation mass flux could then be found by dividing mass difference by sublimation time.

## Results and discussion

### Phase 1: Araneiform formation

**Figure 5:**
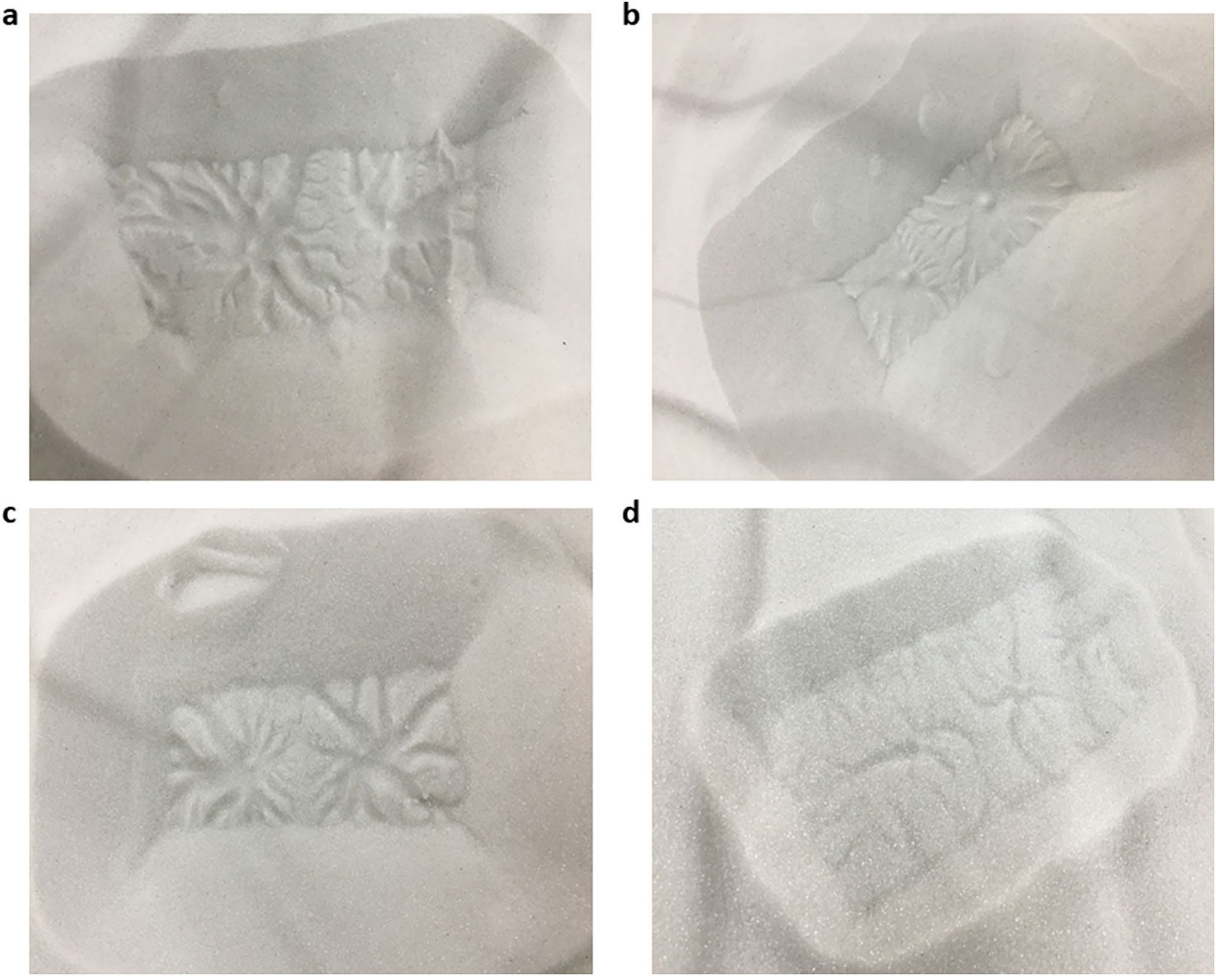
Double araneiforms. Double araneiforms formed on beds of grain sizes ranging from 150 to 600 $\upmu$m when two 3 mm vents were created at the centre of the incident block, 6 cm apart. (**a**) Shows double araneiforms on 150–250 $\upmu$m grains. (**b**) Shows double araneiforms on 150–250 $\upmu$m grains. (**c**) Shows double araneiforms on 250–425 $\upmu$m grains. (**d**) Shows double araneiforms on 450–600 $\upmu$m grains. Sublimation was highly vigorous for the 160–212 $\upmu$m experiments, particularly with two central vents. This caused rapid sediment transport back on top of the block and pit and hence surface features were concealed by this burying effect.

**Figure 6:**
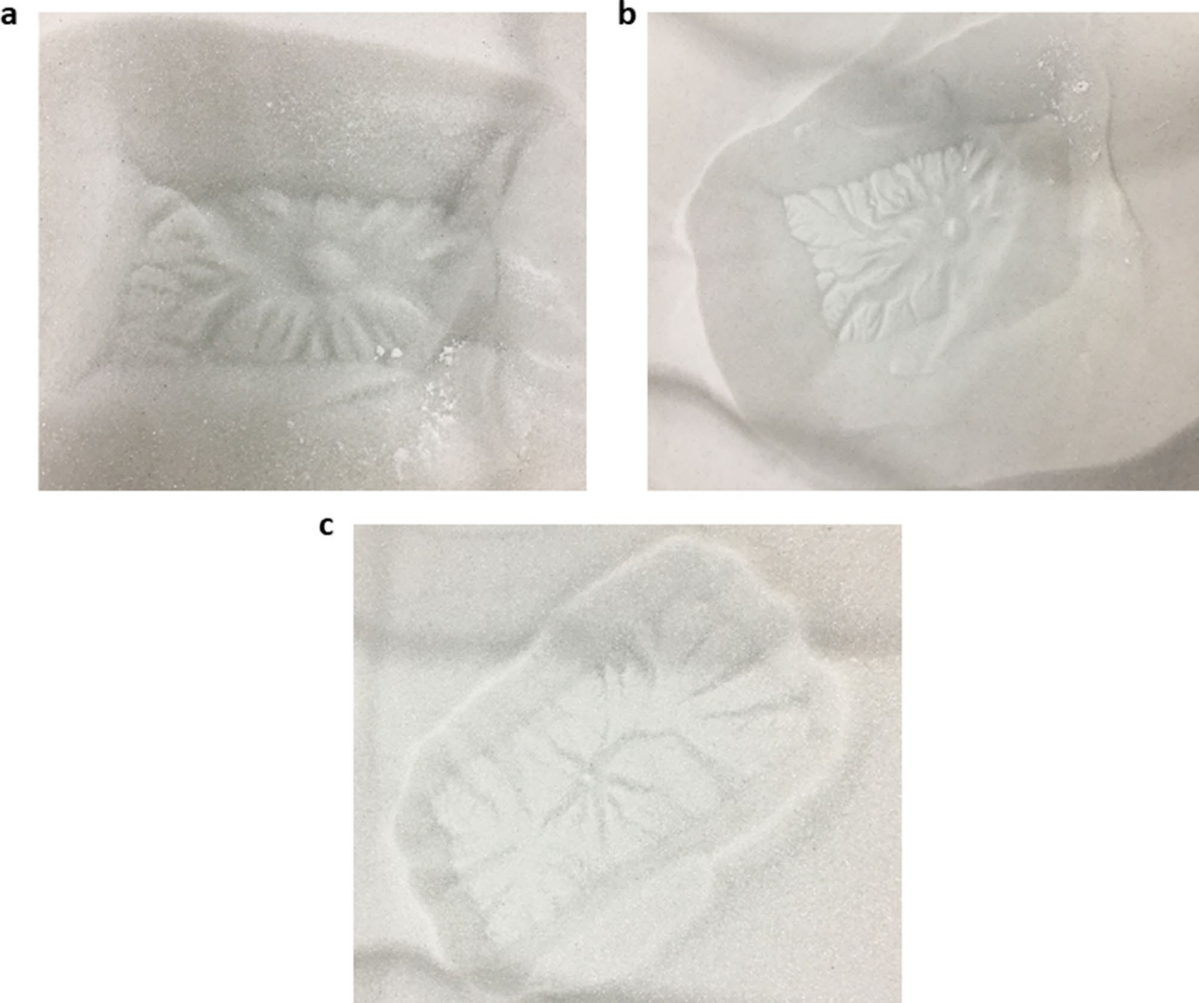
Area covered by araneiform with grain size. (**a**) Shows araneiform formed with a 5 mm vent on 160–212 $\upmu$m grains. (**b**) Shows araneiform formed with a 5 mm vent on 150–250 $\upmu$m grains. (**c**) Shows araneiform formed with a 5 mm vent on 425–600 $\upmu$m grains. The area the araneiforms cover appears to increase with smaller grain sizes.

**Figure 7:**
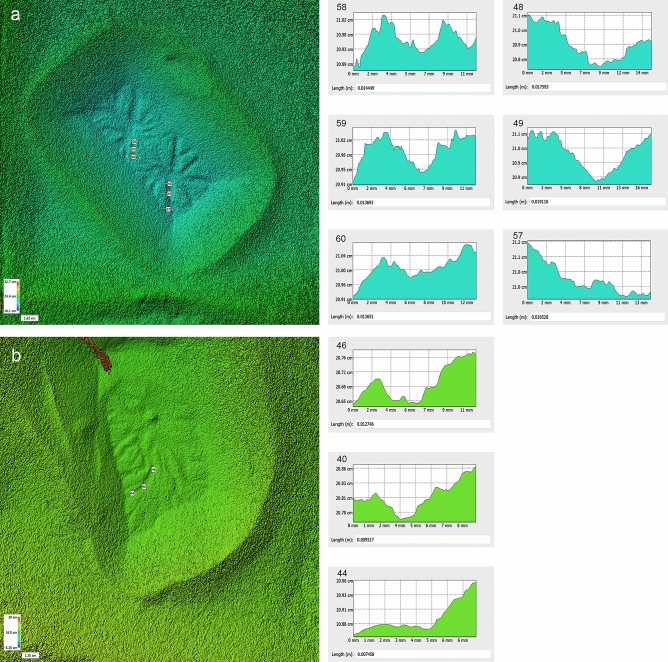
Examples of DEMs created in the laboratory with cross-sections showing depth of troughs decrease away from araneiform centre, as on Mars. (**a**) Shows double araneiforms on 250–425 $\upmu$m grains. (**b**) Shows an araneiform on 150–250 $\upmu$m grains. Graphs show decrease in trough depth further away from the central pit. Sample depths were calculated by averaging the heights of the inversion points at each side of the trough and taking away the minimum point, using linear transects to calculate depth in Agisoft Metashape. Transect numbers are arbitrary. (**a**) Transect 58 was 0.102 cm deep, transect 59 was 0.0859 cm deep, transect 60 was 0.07 cm deep. Transect 48 was 0.189 cm deep, transect 49 was 0.123 cm deep, transect 57 was 0.06 cm deep. (**b**) Transect 46 was 0.071 cm deep, transect 40 was 0.041 cm deep, transect 44 was 0.025 cm deep.

Features analogous in morphology (though orders of magnitude lesser in scale) to araneiforms formed following each experimental trial (Figs. 4, 5, 6). On Mars, araneiforms are between $\sim \,10\,$m and $~\,1\,$km in total extent while our experimental chamber and ice supply limited physical modelling of araneiforms to the cm scale. Numerical modelling will be required to extrapolate the processes observed in the laboratory to the Martian scale. Excluding the vast difference in scale however and considering morphology only, we used the classification for araneiforms provided by Hansen^13^ to identify the features and to subclassify them. Each feature had negative topography, had a central depression and troughs which were dendritic and tortuous, especially for finer grain sizes. Troughs decreased in depth with distance from the central depression (Fig. 7), similar to the Martian forms. In the majority of cases the troughs were radially organised, barring the instances where they appeared to interlink and overlap in a more disorganised ‘lace’ morphology. All radial dendritic patterns formed in one instance and did not require multiple events of trough connection towards a common centre. As the block was uniform in each case, the gas flowed beneath the block to the central vent.

#### Plume observations

Plumes of ${\hbox {CO}}_2$ ejecta entraining sediment were observed in each case (Video 1). These are the first observations of plumes depositing material on top of ice and consequently eroding radial araneiform structures. These types of plumes; driven by momentum but denser than the ambient fluid are known in the fluid dynamic community as fountains. Unlike on Mars where winds are thought to frequently redistribute the jetted subsurface material in fan shapes away from the centre of the araneiform, the material carried within the plume either fell down onto the surrounding bed or directly back down through the vent. The latter process formed a central raised spot within the central depression. Plume height was simply observed and not measured because in some cases, the plume height was limited by the height of the chamber but differences according to (1) vent diameter and (2) grain size, could be quantitatively observed. Vent diameter affected plume height significantly, with ejecta from plumes emanating from 3 mm diameter vents reaching the chamber ceiling ($\sim \,0.6\,$m above the bed surface) in many cases at high velocity relative to plumes from 5 mm vents, while 5 mm vent plumes were more diffuse, with grains only reaching the top of the claw ($\sim \,0.15\,$m above the bed surface). This is to be expected since as we showed earlier the velocity in the vent will be $U=Aq/a$, so if the vent radius decreases from 5 to 3 mm the velocities will increase by $(5/3)^2$ and the height of the resulting granular plume by $(5/3)^4 \approx 8$, since $h \approx gU^2/2$. The larger grains do not reach the gas velocity in the vent and the plume heights are therefore less.

#### The effect of grain size on araneiform morphometry

The effect of grain size on the level of branching of the araneiforms is seen in Fig. 3a. Points where fallen sediment covers part of the araneiform are not included in the plot. The level of branching of araneiforms decreases with increasing grain size. In addition, araneiform legs were observed to be more sinuous for finer grain sizes. Outlying points for 150–250 $\upmu$m are probably due to erasure of part of the eroded pit surface by infalling sediment from levées and from grains from the top of the lifted ice block. As in Table 1, the difference between the widest and thinnest leg of the araneiform became less with increasing grain size also. This is to be expected since for the same flow velocity, the Shields parameter (Eq. 1) is smaller for larger diameter grains; thus they will erode less. Most notable is the difference in morphometry between all other grain sizes and our coarsest grain sizes of 425–600 $\upmu$m which show very low levels of branching. This dependence on mean grain size or sorting may help to explain the distinction between the location of furrows and dendritic troughs and the dendritic araneiform terrain and indeed, other environmental conditions on Mars. Furrows can have a range of morphologies including linear and sinuous, are very shallow^33^ and are only found on dune slopes and sandy patches in interdune regions. An upper limit of the grain size distribution on Martian dunes was recently provided by the Curiosity rover expedition to the Bagnold dune field on Mars from $<\,150\,\upmu m$ to 1 mm^34^. Dendritic troughs are found on interdune material which is believed to be finer and less consolidated, while grains in the SPLD were found to range from 50 to 200 $\upmu$m^15^. Since SPLD grains are in general, finer than those on dune slopes and yet erosion to the degree observed for furrows has not been observed in these regions, it is likely that other environmental factors contribute to lack of erosion, such as sediment consolidation.

**Table 1:** Summary of measured and controlled parameters.

Block area (cm$^2$)	Block mass (g)	Vent diameter (mm)	Grain size ($\upmu$m)	Width centre (cm)	Pit floor area (cm$^2$)	Araneiform area (cm$^2$)	% Area	Widest trough (cm)	Thinnest trough (cm)	DEM resolution (mm/pix)	Orthophoto resolution (mm/pix)	RMS Model uncertainty (m)	Number of junctions
241.5	820	5	425–600	2.69	176.07	14.37	8.16	0.7	0.5	0.619	0.155	0.01	8
234	760	Double	425–600	1.36	78.4	9.07	11.57	0.74	0.46	0.76	0.19	0.002	5
234	760	Double	425–600	1.82	71.38	8.04	11.26	0.72	0.42	0.76	0.19	0.002	5
230	750	3	425–600	1.33	162.23	7.08	4.36	0.8	0.39	0.533	0.133	0.002	9
224.3	750	Double	425–600	1.23	69.46	5.96	8.58	0.91	0.45	0.636	0.159	0.003	5
224.3	750	Double	425–600	1.13	81.23	9.63	11.86	1.17	0.48	0.636	0.159	0.0034	5
209	550	5	425–600	1.13	182.3	19.66	0.11	0.91	0.33	0.524	0.131	0.0037	6
207	710	3	425–600	1.42	168	6.64	3.95	0.64	0.264	0.621	0.115	0.0022	6
214.5	690	5	250–425	5.3	118.7	62.36	52.54	1.02	0.3	0.707	0.117	0.0044	15
240	850	3	250–425	7.4	109.1	28.8	26.4	0.54	0.14	0.497	0.122	0.0042	21
240	830	3	250–425	4.7	134	76.55	57.13	1.77	0.475	0.533	0.133	0.008	10
246	830	Double	250–425	2.9	39.84	21.3	53.46	1.1	0.467	0.414	0.104	0.0058	12
246	830	Double	250–425	2.92	46.55	24.2	51.99	1.07	0.55	0.414	0.104	0.0058	17
203.5	620	5	160–212	6.44	80.4	46.87	58.3	2.04	0.378	0.477	0.119	0.0021	26
203.5	615	5	160–212	7.2	80.4	46.87	58.3	0.95	0.32	0.614	0.154	0.002	28
203.5	600	3	160–212	5.87	100.28	54	53.85	0.91	0.284	0.489	0.112	0.0023	28
224.3	680	5	150–250	4.3	124.42	46.14	37.08	1.44	0.24	0.444	0.111	0.0099	40
240	745	5	150–250	2.93	47	5.1	10.85	0.82	0.12	1.4	0.138	0.0159	28
246	830	Double	150–250	4.7	49.37	40.81	82.66	1.38	0.263	0.524	0.131	0.0044	28
246	830	Double	150–250	4.4	31.05	23.5	75.68	2.02	0.136	0.524	0.131	0.0045	17
224.3	670	Double	150–250	4.1	45.52	24.4	53.6	1.27	0.367	0.629	0.157	0.0022	35
224.3	670	Double	150–250	3.67	66.21	28.87	43.6	1.1	0.387	0.629	0.157	0.0022	30
224.3	790	3	150–250	4.32	127.15	49.46	38.9	1.58	0.575	0.46	0.115	0.0025	36
224.3	750	3	150–250	6.1	120.22	69.66	57.94	1.3	0.3	0.403	0.101	0.0068	45

The effect of grain size on the area covered by araneiforms is seen in Figs. 3b and 6. Finer grains are more mobile and hence the imprint of many ${\hbox {CO}}_2$ subsurface gas conduits are visible on the resultant araneiform (Fig. 6b). There appears to be a cut off point at 425–600 $\upmu$m where while the radial patterns are detectable, these are shallow and have very low levels of branching (Fig. 6c). Araneiform development will of course vary with the vast scale differences between the laboratory setting and the SPLD on Mars and with other external factors such as ice grain size and thickness. However, while vent separation was kept relatively consistent between different grain size trials, we report that the extent to which the radial troughs of araneiforms reach (acknowledging edge effects introduced by the small block size from the sides of the block) is dependent on grain size.

#### The effect of vent diameter on araneiform morphometry

The effect of vent diameter on the level of branching and feature morphometry is subtle for our range of grain sizes. We attribute this to not having wide enough blocks to explore the natural course of araneiform troughs which in some cases are curtailed by vents at the edges of the blocks. However, in the cases where 5 mm vents were used, troughs reach the edge of where the block was placed and the central depressions are noticeably wide in comparison to the length of araneiform troughs (Fig. 4). Additionally, the level of branching for the finest three grain size ranges was generally higher when a smaller 3 mm vent was used.

Because vents were acting at both the sides of the block and at the location of the central hole, in all cases, short, linear or sinuous furrows also formed at the sides of the block as in^26^. This was expected as vents would form at regions where there was a slight gap between the block and the granular surface. In many cases, the araneiform troughs stopped at the location where the edge of the block was, suggesting that in these instances, troughs would extend further had there been more distance to the next vent. Double araneiforms formed when 3 mm vents were acting 60 mm apart (Fig. 5). However, no conclusion can be drawn on the influence of vent separation on araneiform morphometry as our ${\hbox {CO}}_2$ samples were so small. Vent separation in our cases ranged only from 50 to 105 mm. A deeper insight to the effect of vent separation on araneiform morphologies could be achieved by using larger slabs of ${\hbox {CO}}_2$.

Our initial results indicate that there may be a feedback between vent diameter and araneiform morphometry. If their starting radial patterns formed in one venting episode, ‘fat spiders’ may have formed by wider vents or close vent separation, whereas the more dendritic ‘thin spiders’ with small central depressions could have formed by the action of small vents or wide vent spacing. Additionally, given the sharp difference in morphometry with grain size and the fact that the distinct Martian araneiform morphologies tend to cluster in separate regions, it is possible that different levels of regolith consolidation or grain size may influence the different morphologies.

### Phase 2: ${\hbox {CO}}_2$ sublimation efficiency

When a ${\hbox {CO}}_2$ block was allowed to sublimate on beds of 160–212 $\upmu$m and 150–250 $\upmu$m, a vigorous rapid sublimation process was observed (Video 2). In each experiment, the block spun and was highly mobile within the container via sublimating gas exerting pressure on surrounding grains. In each case, the mobility of the block was limited by the floor or walls of the container and it is thought that the block would have continued on its course if not obstructed. Rapid sublimation continued until the temperature of the granular bed reached $\sim \,-30\,^{\circ }$C. Sediment was transported as high as the chamber roof ($\sim \,0.6$ m above the bed surface) and so we report a lower limit of how high sediment can be transported via this process.

If heat flux was purely through solid contacts and radiation there would be little difference between sublimation mass fluxes under Martian and Terrestrial atmospheric conditions for the sand temperatures. However, under Martian conditions since the pressure is more than 100 times lower the same mass flux will give rise to a volume flux that is 100 times larger and thus velocities that are 100 times larger. This will massively increase the erosional capacity of the flow since this scales as $U^2$. In addition the flow is more likely to be turbulent and have a higher turbulence intensity thus increasing heat transport. Thus we do also expect a higher sublimation mass flux of ${\hbox {CO}}_2$ under Martian atmospheric pressure since this is set by the heat transport. In one experiment the mass flux increased by a factor of 16. In each case, sediment was transported out from the container to the surroundings of the chamber. As outlined in “Methods”, the percentage volume of material transported out of the container was calculated in each case. This ranged from 6 to 16% of material ejected from the confinement of the container resulting in $\sim$ 8–27$\%$ of material excavated by ${\hbox {CO}}_2$ sublimation per kg of ${\hbox {CO}}_2$.

Extrapolating these values to the amount of ${\hbox {CO}}_2$ required to mobilise a 1000 m$^3$^13^ volume araneiform on Mars is not informative as the situation involved in the subsurface heating of regolith beneath an ice overburden is vastly different to a buried ${\hbox {CO}}_2$ ice block. The former is a gradual process and not all faces of the ${\hbox {CO}}_2$ layer would be exposed to warm regolith. Additionally, the scaling differences in sediment ejecta distances are significant, as well as the differences in $\Delta$T compared to Mars. The extreme difference in ice mass and thickness will introduce factors which affect sublimation efficiency, as well as differences due to lower gravity on Mars. A detailed numerical model will be required to exploit the data reported here in order to further understand the vast scale of the araneiforms of the SPLD on Mars. Modelling will provide a better understanding of the energy budget to erode these large-scale patterns in few instances under a different climate. However, we have provided a first step in empirically investigating the efficiency of sublimating ${\hbox {CO}}_2$ ice as an agent of the transport of granular material. Both the qualitative (observational) and quantitative data may be drawn upon in order to test, refine and develop models of ${\hbox {CO}}_2$ sublimation under Martian conditions; for example, ejecta volumes per mass ${\hbox {CO}}_2$ may elucidate whether the Recurring Diffusive Flows (RDFs) recently observed surrounding linear dune gullies^35^, are darker albedo subsurface material ejected laterally via sublimation as ${\hbox {CO}}_2$ blocks slide down dune slopes and bury at their termini.

**Figure 8:**
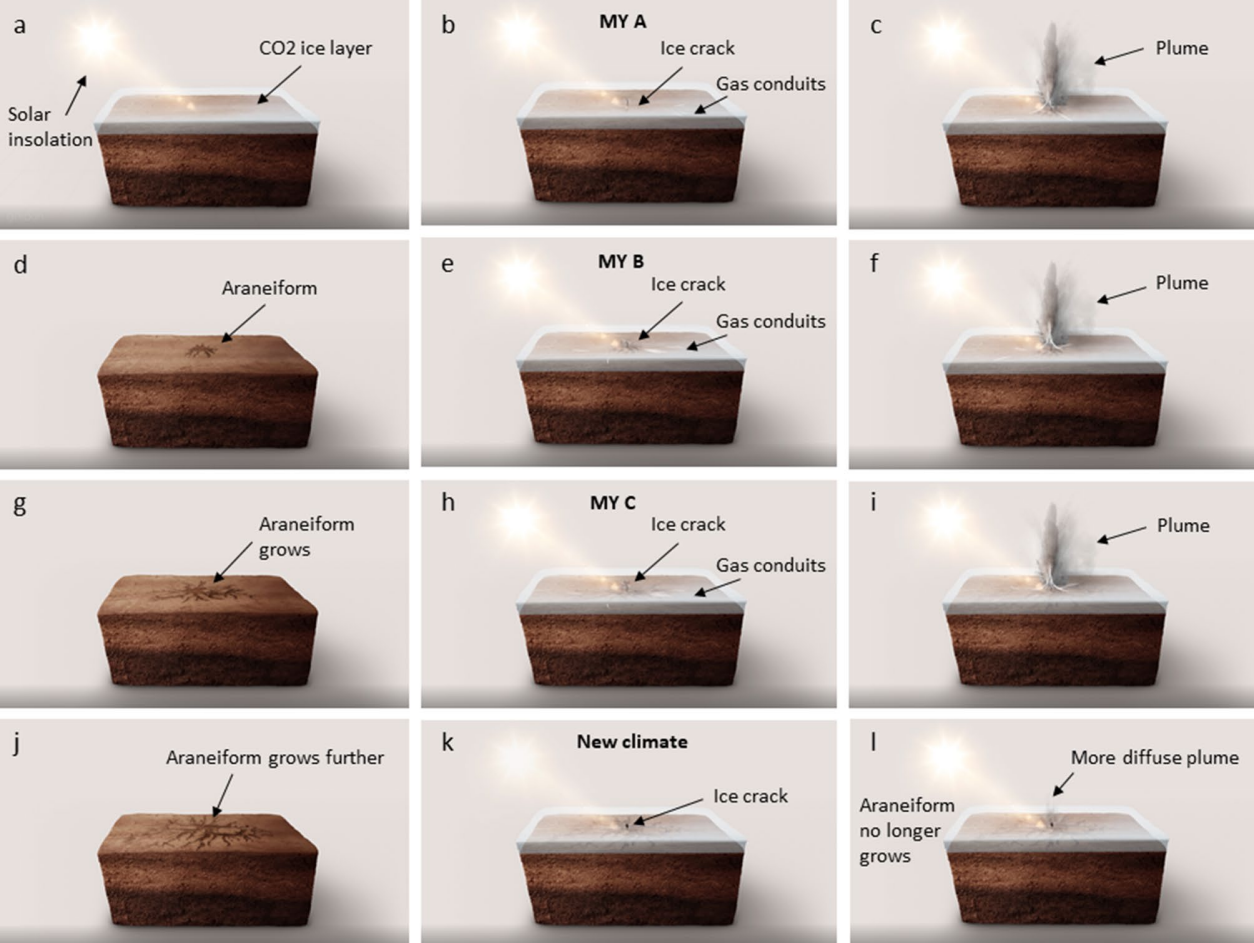
Araneiform development hypothesis suggesting araneiforms formed in a paleoclimate and their activity today is not growing existing radial araneiforms. (**a**–**d**) Show initial araneiform development as suggested by Kieffer and Piqueux^3, 9^. (**e**–**j**) However we suggest that a radial structure develops initially and grows each MY during this paleoclimate which is conducive to more energetic cryoventing. (**k**, **l**) Show the present day Martian climate where fans and spots still reappear, however we suggest the terrain is not eroded and fans and spots are recycled dust transported by a much more diffuse plume than in (**c**, **f**, **i**). Source: Collaboration between Wax Visuals and LEM. Wax visuals created the artistic representation of the araneiform development hypothesis.

Nonetheless, we have shown that radial araneiform *patterns* in the laboratory can begin in one venting event. From our observations that on the lab scale at least, araneiforms can begin as one radial structure, and erosion efficiency appears to depend on grain size and vent width, we hypothesise that it may be possible for Martian araneiforms to begin their lifetime as a radial pattern which eventually grew outward by successive events, perhaps in a climate more conducive to more energetic sublimation with more intense insolation/higher temperatures (Fig. 8a–d) during the planets changes in obliquity, or when regolith was less consolidated. If the radial patterns of Martian araneiforms did indeed form in a past climate, it is possible that that a smaller scale radial pattern was carved and troughs eventually widened, lengthened and deepened over time to form the giant structures visible on Mars today (Fig. 8g,h). As previous authors have suggested^22^, it is possible that the existing topography provided by the araneiforms causes points of weakness (Fig. 8d) whereby overlying ice is more likely to crack seasonally. However, the underlying material may be too consolidated to be eroded further today or present day climate conditions are not conducive to the energy required to erode coarser or more consolidated granular material. It is plausible that the dark fans and spots which appear annually in similar locations are therefore recycled dust which deposits in autumn and settles into the negative topography of the araneiforms. Only 7 MY have been monitored by HiRISE and limited temporal studies have been conducted^2^. The upcoming extended Planet Four: Terrains^2,11,12^ campaign results, along with extensive numerical modelling and comparison with predicted past climatic conditions from Global Climate Models are opportunities for further insights on whether this is the case, or whether the araneiform troughs continue to grow over time.

## Conclusion

We have reported the first physical observations of (1) araneiform formation and (2) plume activity and have reconciled feature with process on the laboratory scale. We show that sublimation rates under Martian pressure can be an order of magnitude larger than those on Earth due to enhanced turbulent heat transport. We find that the area covered by araneiforms on the laboratory scale decreases with increasing grain size suggesting that regions of different regolith consolidation or grain size on Mars might constrain the varied araneiform morphometries. We report that the level of branching of araneiforms increases with (1) decreasing grain size and (2) smaller vents, lending further credence to the proposal that this is governed by the Shields parameter. We find that wider centres result from wider vents and that shorter, more consistent width troughs are formed on coarser grain sizes, suggesting that perhaps the ‘fat’ spider morphologies are generated at lower Shields parameters and that classic, ‘thin’ spiders form with smaller vents or finer grains at higher Shields parameters. We show that radial dendritic araneiform patterns can form in one venting process and at this scale, do not require multiple events to cause singular dendritic structures to connect at a central point. We offer an alternative hypothesis that radial araneiform patterns developed in one or few instances in a past climate and that perhaps trough dimensions grew over multiple MY. We suggest rigorous temporal surveys of araneiforms over multiple MY will shed light on whether dust ejecta originate from the troughs or are merely seasonal dust deposits which are recycled annually in this unique geomorphic process which is unlike any on Earth.

## Supplementary information

Supplementary Information 1.

Supplementary Information 2.

Supplementary Video 1.

Supplementary Video 2.

Supplementary Information 3.

Supplementary Information 4.

Supplementary Information 5.
