# Supplementary material for: The formation of araneiforms by carbon dioxide venting and vigorous sublimation dynamics under martian atmospheric pressure
Source: Sci Rep. 2021 Mar 19;11:6445. doi: 10.1038/s41598-021-82763-7 (PMC7979800; doi:10.1038/s41598-021-82763-7)
Supplement: Supplementary file 5 — Supplementary Information 3. [file 41598_2021_82763_MOESM5_ESM.pdf]

# Supplementary Material: The Formation of Araneiforms by Carbon Dioxide Venting and Vigorous Sublimation Dynamics Under Martian Atmospheric Pressure


Mc Keown, L. E. [1,5], McElwaine, J. N. [2,3], Bourke, M. C. [1], Sylvest, M. E. [4], & Patel, M. [4]

[1] *Trinity College Dublin, College Green, Dublin 2, Ireland*

[2] *Durham University, Durham DH1, UK*

[3] *Planetary Science Institute, 1700 E Fort Lowell Rd, Tucson, AZ 85719, USA*

[4] *The Open University, Walton Hall, Kents Hill, Milton Keynes MK7 6AA, UK*

[5] ***Now at** The Natural History Museum, Cromwell Road, South Kensington, London SW7 5BD*


**Supplementary Videos**

- Video 1: Video showing a plume of $CO_2$ gas and sediment ejecta emanating from a central 5 mm vent on a bed of 150-250 $\mu m$ grains.
- Video 2: Video showing a violent, rapid sublimation process when a $CO_2$ block was placed on a bed of 150-250 $\mu m$ grains.
